# Retinal microvascular changes in white matter hyperintensities investigated by swept source optical coherence tomography angiography

**DOI:** 10.1186/s12886-021-02143-7

**Published:** 2022-02-15

**Authors:** Yuzhu Gao, William Robert Kwapong, Yifan Zhang, Yuying Yan, Xurui Jin, Yunhan Tao, Hanyue Xu, Bo Wu, Ming Zhang

**Affiliations:** 1 Department of Ophthalmology, West China Hospital, Sichuan University, No.37 Guoxue Lane, Chengdu, Zip code: 610041 Sichuan Province China; 2 Department of Neurology, West China Hospital, Sichuan University, No.37 Guoxue Lane, Chengdu, Zip code: 610041 Sichuan Province China; 3 Duke Global Health Institute, Duke University, Durham, NC USA

**Keywords:** White matter hyperintensities, Retina, Foveal avascular zone, Swept source optical coherence tomography angiography, Microvascular

## Abstract

**Backgro:**

To assess the microvascular changes in the macular region and the foveal avascular zone (FAZ) area in participants with white matter hyperintensities (WMHs) using swept source optical coherence tomography angiography (SS OCTA).

**Methods:**

This cross-sectional study included a total of 23 WMH participants (45 eyes) and 20 age-matched healthy participants (40 eyes). SS OCTA (VG200; SVision Imaging, Ltd., Luoyang, China) was used to assess the retinal vessel density (VD) and the FAZ area. VD was measured in the superficial vascular plexus (SVP), intermediate capillary plexus (ICP) and deep capillary plexus (DCP) within a 6 × 6-mm scan centred on the macula using a 5-mm Macula circle. The FAZ area was automatically measured on the inner retina layer within a 3 × 3-mm scan in the macular region.

**Results:**

There was no significant difference in VD in the SVP between the two groups. However, VD in both the ICP and DCP was significantly decreased in WMH participants (*P* = 0.028, *P* = 0.016). The FAZ area was significantly enlarged in WMH participants (*P* = 0.030). The signal quality was significantly lower in WMH participants (*P* < 0.001).

**Conclusions:**

This study suggested that WMH participants have retinal microvascular and foveal avascular zone area changes compared with healthy controls. Further longitudinal studies with larger sample sizes are warranted to identify the value of our findings in the early evaluation of WMHs.

## Background

Alzheimer’s disease (AD) is the most common cause of dementia and has been reported to affect millions of elderly individuals worldwide [1, 2]. A loss of neurons, atrophy of the brain and deposition of amyloid plaques are normally associated with the pathophysiologic process of AD [3]. Nonetheless, the classic symptoms of AD, progressive memory loss and changes in behaviour, are recognized as occurring or manifesting after considerable, irreversible neuroaxonal loss in the brain [4, 5]. Thus, clinicians have suggested that the early phase or preclinical phase of AD may be the new route to help develop timely and effective diagnostic approaches to help slow the progression of the disease. Furthermore, with such tools, early-stage targeted intervention could be conducted to prevent AD.

White matter hyperintensities (WMHs), as measured on magnetic resonance imaging (MRI), are normally found in the ageing population and are characterized as hyperintensities on T2-weighted or flair-attenuated inversion recovery (FLAIR) images. Reports have shown that WMHs are found in over 50% of older adults [5, 6] and are associated with the development of AD and dementia [7, 8]. Although the pathogenesis and clinical relevance of WMHs are still vague, recent studies have suggested that WMHs are marked by microvascular dysfunction in the cerebral microcirculation [9], and some pathological studies have suggested that cerebral small vessel changes occur in WMH participants. In addition, some cerebral imaging studies found significant neuronal loss in WMH participants [10, 11]. However, whether microvascular dysfunction contributes to neuronal loss in the brain is unclear. It is vital to be able to detect the association between them.

Optical coherence tomography (OCT) is a well-tolerated, noninvasive imaging modality that provides high-resolution images of the retina and has been widely applied in numerous fields, especially ophthalmic imaging [12, 13]. Recent reports have suggested that retinal microcirculation could be a useful tool to evaluate cerebral microcirculation [14]. Previous retinal imaging reports have found venous narrowing and reduced blood flow in WMH participants compared with healthy controls [15–17]. A study using optical coherence tomographic angiography (OCTA) evaluated the retinal microvasculature in WMH participants and suggested that decreased deep capillary density was associated with WMHs [18]. However, detailed information on the capillary plexus of the macula and the foveal avascular zone (FAZ), an important indicator of AD, was not studied in previous reports. Swept source optical coherence tomographic angiography (SS OCTA) is a new and improved noninvasive retinal imaging modality that can help to visualize and evaluate the in-depth and deepest capillaries of the macula. Hence, SS OCTA may enable quick, inexpensive, and noninvasive screening of WMHs.

We hypothesized that the changes in the retina may reflect similar changes in the brain in WMH participants. Accordingly, we used SS OCTA to assess the macular vessel density (VD) and FAZ area in WMH participants and healthy control participants. Additionally, we also investigated whether the SS OCTA tool has the potential to characterize the retinal microvasculature in WMHs.

## Materials and methods

### Study participants

Participants with confirmed WMHs and age-matched healthy participants were recruited in this cross-sectional study. All the WMH participants were dementia- and stroke-free but had white matter lesions on MRI (Fazekas score greater than 1 but less than or equal to 3) and in the subcortical region of the brain. The inclusion criteria for the WMH participants were as follows: 1) aged over 50 years; 2) could cooperate during MRI and retinal imaging; and 3) Chinese. The exclusion criteria were as follows: 1) eyes with known ocular diseases such as glaucoma and age-related macular degeneration; 2) a refractive error of less than − 6.0 dioptres and prior intraocular surgery; and 3) systemic disease that might affect eyes such as systemic lupus erythematosus and diabetes mellitus.

All participants underwent standard ophthalmic examination, including best-corrected visual acuity (BCVA) testing using the Snellen chart, slit-lamp biomicroscopy, intraocular pressure (IOP) measurement, and dilated fundus examination. Moreover, for WMH participants, Fazekas scores were obtained and MRI was performed for evaluation.

This study was approved by the Institutional Review Board for Human Research at West China Hospital, China, and conducted in compliance with the Declaration of Helsinki. All enrolled participants provided written informed consent.

### Optical coherence tomography angiography imaging and image processing

All participants were imaged with SS OCTA with a scanning speed of 200,000 A scan per second, widefield angle of 56 degrees, and an imaging depth of 2.7 mm in tissue at 1050 nm (VG200; SVision Imaging, Ltd., Luoyang, China). Both 3 × 3 and 6× 6-mm scans were performed in the centre of the macula three times for each participant, which consisted of 512 horizontal A lines at 512 vertical locations with four repeated scans in each fixed location, resulting in a sampling spacing of 12 μm.

The presence of blood vessels is directly indicated by angiography signals. An algorithm is designed to separate the foreground (blood vessel) pixels from background (non-vessel tissue) pixels by properly segmenting the image from the perspective of angiography signal strength. The VD is defined as a metric used to describe the percentage of area occupied by blood vessels in a two-dimensional retina projection image. The projection image is acquired by projecting three-dimensional angiography volume data onto a two-dimensional imaging plane, which is also called an en face image. As described, then the ratio of blood vessel pixels as a percentage of all pixels is computed within a pre-defined window size.

**Fig. 1:**
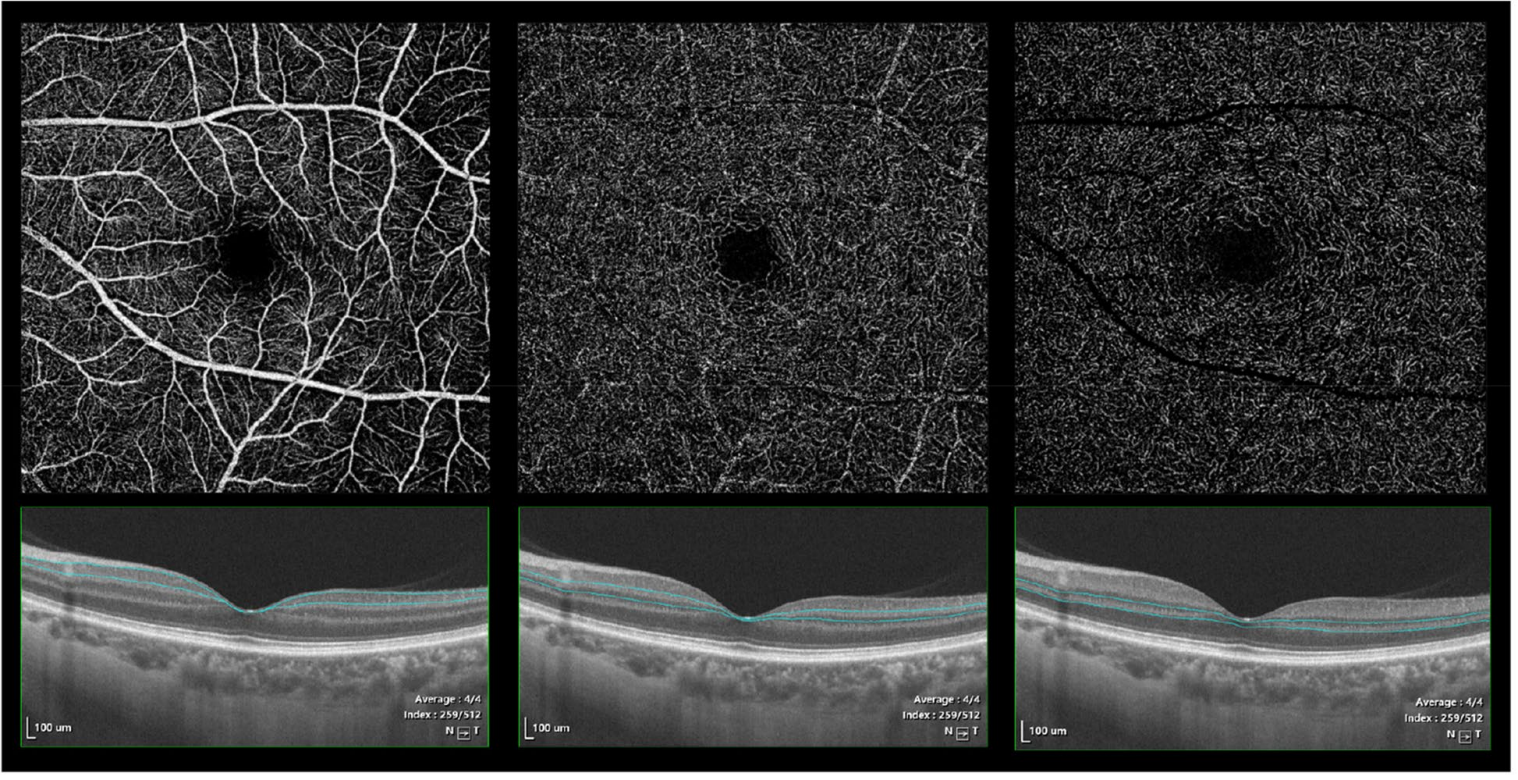
En face SS OCTA images of three layers participates with corresponding segmenting slabs: **A** superficial vascular layer **B** intermediate capillary layer **C** deep capillary layer (from a WMH participant)

OCTA images selected with good scan quality (more than 7/10 signal strength as defined by the manufacturer) and without severe motion artefacts were included. Retinal layers were segmented using a validated semiautomated segmentation algorithm and manually calibrated by two experienced ophthalmologists for accuracy. The superficial vascular plexus (SVP), the intermediate capillary plexus (ICP) and the deep capillary plexus (DCP) were automatically segmented by the software of the device. The SVP slab was segmented as the inner 80% of the ganglion cell complex (GCC) (defined as the nerve fibre layer (NFL) + ganglion cell layer (GCL) + inner plexiform layer (IPL)), excluding the NFL. The intermediate capillary plexus (ICP) was generated from the outer 20% of the GCC to the inner 50% of the inner nuclear layer (INL), the deep capillary plexus (DCP) was generated by a slab extending from the outer 50% of the INL to 25 μm beneath the interface INL/outer plexiform layer (OPL), and the inner retina extended from 5 μm above the interface ILM to 25 μm beneath the interface INL/OPL (Fig. 1).

**Fig. 2:**
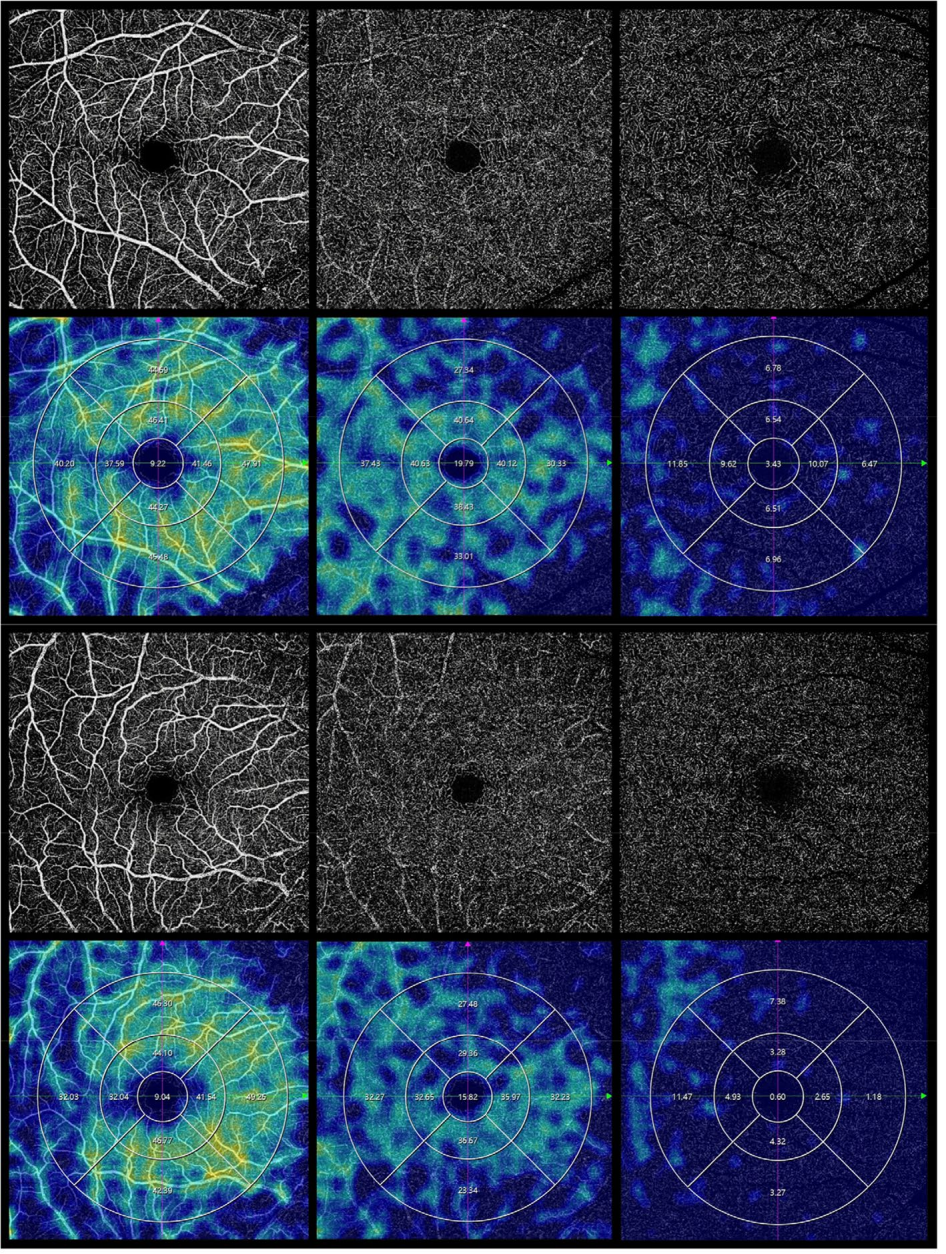
Representative SS OCTA 6 × 6-mm image of vessel density of superficial vascular layer, intermediate capillary layer and deep capillary layer centred on macula, the vessel density decreased significantly in the WMH participant: **a** from a healthy control participant **b** from a WMH participant

**Fig. 3:**
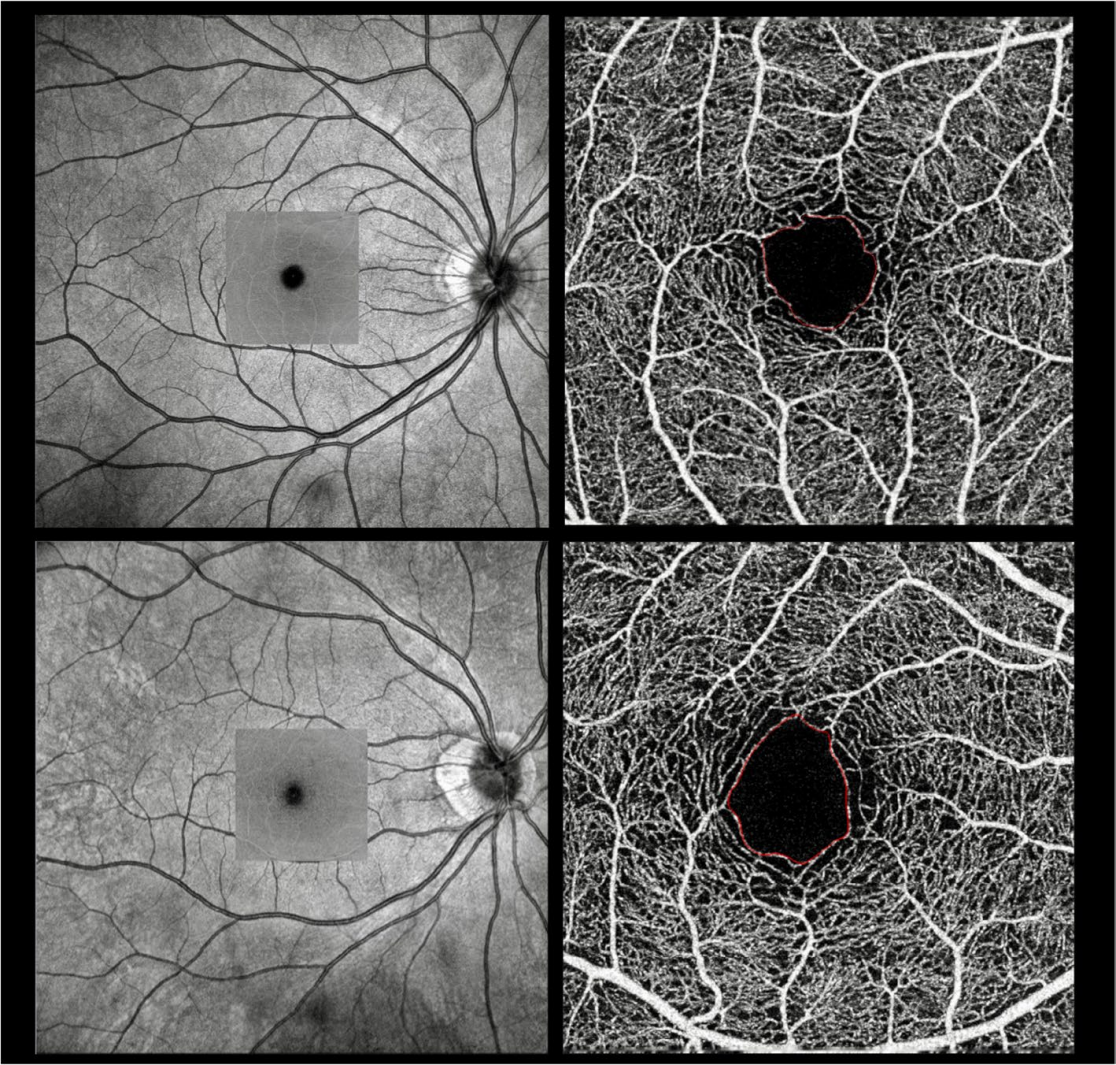
Representative SS OCTA 3 × 3-mm images of inner retina layer and delineation of the FAZ: **a-b** from a Healthy control participant **c-d** from a WMH participant

The VD was calculated according to the 5-mm Macula-ring circle on the SVP, ICP and DCP centred on the macula from a 6 × 6-mm scan and assessed by the mean value of the 9 regions (Fig. 2). The FAZ area was automatically measured based on en face images of the inner retina layer from a 3 × 3-mm scan (Fig. 3).

### Statistical analysis

Baseline characteristics are presented as the mean (continuous variables) or frequency distribution (categorical variables). The Shapiro-Wilk test was used to test the normality of the data. Generalized estimating equation (GEE) models were used to compare the differences in microvascular density while adjusting for risk factors and inter-eye dependencies. A *P* value less than 0.05 was considered statistically significant throughout the study. Statistical analysis of collected data was performed using IBM SPSS Statistics version 23 (SPSS Inc., Chicago, IL, USA).

## Results

A total of 23 WMH participants and 23 age-matched healthy control participants were recruited. Three healthy control participants were excluded owing to unpredictable drusen during the checking procedure. Thus, 45 eyes from 23 WMH participants and 40 eyes from 20 healthy control (HC) participants were enrolled in imaging processing. Baseline characteristics are shown in Table 1. In all 23 WMH participants, these white matter lesions were in the subcortical portion of the brain. The mean age of the WMH participants was 59.14 ± 5.75 years, and that of the healthy control participants was 56.37 ± 3.55 years. There were no significant differences between groups in sex (with WMHs, 15 males and 8 females; 9 males and 11 females in healthy controls), IOP (with WMHs, 13.74 ± 1.03; 14.09 ± 2.13 of healthy controls) or axial length (with WMHs, 22.94 ± 9.01; 23.03 ± 7.20 of healthy controls). The average LogMAR visual acuity was 0.13 ± 0.16 in the WMH group and − 0.01 ± 0.12 in the healthy control group (*P* < 0.001). The mean Montreal Cognitive Assessment (MoCA) score was 25.14 ± 1.29, and the mean Fazekas score was 2.2 ± 0.45 in the WMH group.

**Table 1:** Baseline Characteristics

	WMH	HC	*P* value
Number	23	20	
Number of eyes	45	40	
Gender (M:F)	15:8	9:11	0.498
Age, years	59.14 ± 5.75	56.37 ± 3.55	0.068
IOP, mmHg	13.74 ± 1.03	14.09 ± 2.13	0.396
Axial length, mm	22.94 ± 9.01	23.03 ± 7.20	0.821
VA, LogMAR	0.13 ± 0.16	−0.01 ± 0.12	< 0.001
Hypertension, number	6	0	
MoCA score	25.14 ± 1.29		
Mean Fazekas score	2.2 ± 0.45		
Location of WMHs			
Subcortical, n	23		

Abbreviations: *WMH* White Matter Hyperintensities, *HC* Healthy control, *IOP* Intraocular Pressure, *VA* Visual Acuity, *MoCA* Montreal Cognitive Assessment

Quantitative measurements of VD in the SVP, ICP and DCP and FAZ area are shown in Table 2. Comparing the WMH group to the healthy control group, there was no significant difference in VD in the SVP (*P* = 0.521). However, the WMH group had significantly decreased VD in the ICP (*P* = 0.028) and DCP (*P* = 0.016) in the 5-mm Macula circle compared to the healthy control group. There was also a significant difference in FAZ area in the inner retina between the WMH and healthy control groups (*P* = 0.030). Moreover, the signal quality was significantly higher in the healthy control group than in the WMH group (*P* < 0.001).

**Table 2:** Comparison of vessel density in three layers and the foveal avascular zone area in inner retina

	WMH	HC	*P* value
SVP, %	39.31 ± 3.94	40.13 ± 5.69	0.521
ICP, %	31.34 ± 4.79	34.12 ± 4.96	0.028
DCP, %	9.08 ± 4.10	10.82 ± 3.24	0.016
FAZ, mm^2^	0.40 ± 0.12	0.31 ± 0.08	0.030
Signal quality	7.93 ± 0.65	9.55 ± 0.50	< 0.001

Adjusted for age, gender, signal quality, hypertension, axial length and eyes

Abbreviations: *WMH* White Matter Hyperintensities, *HC* Healthy control, *SVP* superficial vascular plexus, *ICP* intermediate capillary plexus, *DCP* deep capillary plexus, *FAZ* foveal avascular zone area

## Discussion

In this study, we used SS OCTA to investigate the retinal microvasculature and the FAZ area in WMHs and healthy control participants. Our results showed significantly decreased VD in both the ICP and the DCP and an enlarged FAZ area in WMH participants compared with age-matched healthy control participants. These differences suggested that the FAZ area and the VD in the ICP and DCP may provide an earlier marker in screening for WMHs and the preclinical phase of AD.

Compared with healthy control participants, we found significantly decreased VD in both the ICP and the DCP of the retina in WMH participants. Peng and colleagues [18] compared the microvascular changes in WMH participants with different degrees of lesions and indicated that microvascular impairment was associated with the disease cascade in WMHs. Mutlu and colleagues [19] have also reported an association between retinal neurodegeneration and cerebral atrophy, indicating the role retinal OCT plays in providing information on neurodegeneration in the brain.

However, the mechanism of decreased microcirculation in WMHs is still unknown and needs further study. Previous studies have suggested that the changes in the retina reflect the similar ﻿pathological changes occurring in the brain [20], since the retina shares many similarities with the brain, including embryological origin, microvasculature and neuronal projections [21]. It is known that retinal vascular networks are composed of several layers that differ in location, composition and function. The SVP includes flow from the ILM to the middle of the IPL and mainly supplies the ganglion cell layer. It is a network and contains both large and small vessels [22]. The deep vascular plexus (DVP) has two subcomponents: the ICP, containing a slab extending from the IPL to the INL; and the DCP, extending from the INL to the OPL. The ICP and DCP are mainly composed of smaller vessels and capillaries [22]. Since the SVP mainly contains larger vessels and the DVP (ICP and DCP) is related to the function of capillary microcirculation, in our study, the significant decreases in the ICP and DCP may indicate the early appearance of microcirculation dysfunction in the retina in WMH participants. Thus, as a previous report suggested that WMHs may originate from ﻿chronic small vessel ischaemia [23], our study showed that what is seen in the retinal microcirculation may be a reflection of the changes in cerebral microcirculation. Additionally, morphologic substrate, induced by impaired diffusion, was found through thickened vessel walls in WMH participants [24]. Since those changes likely start with smaller vessels, it is of great possibility that those substrates may be present in the small vessels of the ICP and DCP, which may result in early vessel dropout and microvascular dysfunction.

Another possible explanation for decreased VD in the ICP and DCP may be alterations in oxygen. Previous retinal oxygen measurement experiments in animal models have demonstrated that for the inner retina, the dominant oxygen consumers may be located in the plexiform layers (IPL and OPL), probably in mitochondria-rich synapses [25, 26], indicating the need for a highly oxygenated blood supply. Notably, according to Hagag and colleagues [27], in healthy subjects, when compared with other capillary layers of retina, the plexiform layers may maximally constrict under the exposure to hyperoxia, which indicated that the plexiform layers may be influenced greatly under the hypoxic status. Hence, the impaired VD in the ICP and DCP may also be a reflection of the mitochondrial dysfunction and high oxygen consumption found in the brains with WMHs.

We found a significant enlargement of the FAZ area in WMH participants, which has rarely been reported in previous studies. The FAZ refers to the capillary-free area centred on the macula. In this study, the FAZ area was automatically measured in the inner retina, which extends from 5 μm above the interface ILM to 25 μm beneath the interface INL/OPL. Quantifying FAZ parameters has been a useful tool in evaluating macular microcirulation [28]. Since a prior study showed a significant age effect on the FAZ area [29], we selected age-matched participants as controls. The mechanism underlying the enlarged FAZ area in WMH participants remains unknown, but microcirculation dysfunction has been reported to be a cause. Another possible explanation may be neurodegenerative changes. Partial loss of myelin, axons, and oligodendroglion cells in the brain is a common finding seen in WMH participants. The enlargement of the FAZ area in the inner retina may be secondary to both the impairment of neurons and glia in the retina and their effects on the decrease in flow density. Third, perivascular tissue changes, considered the prevailing morphologic substrate [30], may also be a potential explanation. According to Ma KC and colleagues [31], perivascular oedema in the retina, due to intermittent disruption of the blood-brain barrier, could also lead to damage in VD, which results in enlargement of the FAZ area. Hence, the enlarged FAZ area may indicate the impairment of microcirculation in the brain.

Previous studies demonstrated that retinal microvascular density might be associated with axial length [32], sex [33] and refractive errors [34]. However, some remain disputable. Wen and colleagues [32] found that the axial length was negatively associated with superficial parafoveal vessel density but not correlated with VD around the optic nerve head and the FAZ area, while some previous studies indicated that the axial length was negatively correlated with the FAZ area [35]. Milani and colleagues [34] reported that eyes with high myopia were negatively related to superficial VD and positively associated with blood flow of the outer retina. Hence, we adjusted for these parameters during the evaluation.

We acknowledge several limitations in this study. The first is the limited sample size; however, we have used. Second, limited by our study design, we were not able to determine whether these results are related to individual differences. Therefore, we could not be able to determine whether our findings could be translatable to all WMH individuals. However, we designed strict recruitment and made adjustments for multiple comparisons. Further studies with larger sample sizes and longer follow-ups are needed. Third, all the participants were Chinese, which may make our findings hard to generalize to other ethnic groups. The fourth limitation is that we did not measure the microstructural volume of WMH participants. Moreover, most of our WMH participants were in the early stage of the disease; hence, we did not group people according to the severity of disease. Further studies with more detailed neuroimaging data and patients in different disease stages are warranted to enhance our findings and provide in-depth meaning.

There are also some strengths in our study. First, we used SS OCTA for evaluation. The unprecedented scanning speed, imaging depth and sensitivity of SS OCTA improved the accuracy of images. Second, we detected both VD and the FAZ area in WMH participants, which has rarely been reported before and may provide more possible imaging targets for the early diagnosis of WMHs.

## Conclusions

In conclusion, our study showed that SS OCTA may be a new, quick, noninvasive, well-tolerated and inexpensive tool for screening WMH participants based on decreased VD and enlarged FAZ areas. Further longitudinal studies with larger samples are warranted to determine whether our findings have potential meanings in the early detection of WMHs; hence, these participants could be able to receive early care and treatment.

## Data Availability

The datasets used and/or analyzed during the current study available from the corresponding author on reasonable request.

## Abbreviations

AD: Alzheimer’s disease
WMHs: White matter hyperintensities
MRI: Magnetic Resonance Imaging
FLAIR: Flair-attenuated inversion recovery
OCT: Optical coherence tomography
OCTA: Optical coherence tomographic angiography
FAZ: Foveal avascular zone
SS OCTA: Swept source optical coherence tomographic angiography
VD: Vessel density
IOP: Intraocular pressure
SVP: Superficial vascular plexus
ICP: Intermediate capillary plexus
DCP: Deep capillary plexus
GCC: Ganglion cell complex
NFL: Nerve fibre layer
GCL: Ganglion cell layer
IPL: Inner plexiform layer
INL: Inner nuclear layer
GEE: Generalized estimating eq.
HC: Healthy control
VA: Visual Acuity
MoCA: Montreal Cognitive Assessment
